# Non-headache symptoms in migraine patients

**DOI:** 10.12688/f1000research.12447.1

**Published:** 2018-02-14

**Authors:** Ping-Kun Chen, Shuu-Jiun Wang

**Affiliations:** 1 School of Medicine, China Medical University, Taichung, Taiwan; 2 Bo-Zhi Neurology Clinic, Taichung, Taiwan; 3 Department of Neurology, China Medical University Hospital, Taichung, Taiwan; 4 School of Medicine, National Yang-Ming University, Taipei, Taiwan; 5 Brain Research Center, National Yang-Ming University, Taipei, Taiwan; 6 Department of Neurology, Neurological Institute, Taipei Veterans General Hospital, Taipei, Taiwan

**Keywords:** migraine, visual disturbance, allodynia, restless legs syndrome, depression

## Abstract

Migraine is one of the most common neurological disorders. In addition to severe headaches, non-headache symptoms associated with migraine attacks as well as co-morbid disorders frequently aggravate the disabling of migraine patients. Some of these symptoms are related to poor outcomes. In this review, we update the advances of studies on certain non-headache symptoms, including visual disturbance, gastrointestinal symptoms, allodynia, vestibular symptoms, and symptoms of co-morbid restless legs syndrome and psychiatric disorders.

## Introduction

Migraine is a very common neurological disorder characterized by recurrent episodes of disabling headache. In addition to headaches, there are many different non-headache symptoms during or between migraine attacks. These non-headache symptoms of migraine also impact patients’ daily activities as well as their quality of life. In this brief review, we focus on the recent findings of several common non-headache symptoms of migraine, including visual disturbance, gastrointestinal symptoms, allodynia, vestibular symptoms, and symptoms of co-morbid restless legs syndrome (RLS) and psychiatric disorders. Of note, there are a variety of other symptoms, such as phonophobia, neck pain, cognitive dysfunction, and fatigue, which are not discussed in this review.

## Visual disturbance

Visual disturbance is common in patients with migraine. It is an important premonitory symptom: 32% of patients experience light sensitivity and 21% have difficulties in focusing vision ^1^. Photophobia and visual aura are two major types of visual disturbance. Photophobia is more persistent, whereas visual aura is more transient and discrete. In fact, photophobia may begin in the premonitory phase and persist throughout the migraine attack. Up to 80% of migraine patients have experienced photophobia during an attack ^2^. In addition, visual aura is the most common aura and usually occurs in isolation, whereas the other forms of aura usually occur with visual symptoms ^3^. Actually, different types of aura, which may reflect the activation of different parts of the brain (for example, visual and sensory aura), could start at the same time or in succession ^4^. In a Brazilian study on 122 migraine with aura patients, the most common visual symptoms were blurred vision (54.1%) and small bright dots (47.5%), whereas the frequencies of classic zigzag or jagged lines and the typical “C” crescentic shape were 41.8% and 16.4%, respectively ^5^. Hansen *et al*. analyzed more than 1,000 attacks of migraine aura without headache in a single patient for 18 years. The classic aura initially propagated predominantly in the central visual fields and then spread to upper and temporal fields on both sides ^6^. They also found that the visual auras disappeared for several minutes before reappearing in a distant location. In this patient, the auras propagating from V1 to V2 regions of the occipital cortex were suspected to correspond to the visual percept from a scintillating wave to a scotoma ^6^.

Visual snow is very rare and is described as a persistent disturbance in the entire visual field similar to the noise of an analogue television. It is frequently reported to be persistent at all times when eyes are both open and closed. Patients with visual snow were often diagnosed as having persistent visual aura. However, visual snow is frequently concomitant with migraine but usually not responsive to standard migraine treatment ^7^. In addition, patients with visual snow comorbid with migraine frequently have other visual symptoms such as photopsia, nyctalopia, and palinopsia ^7, 8^. In an [18F]-flurodeoxyglucose PET study, hypermetabolism of the right lingual gyrus and the anterior lobe of the left cerebellum were noted in patients with visual snow but not seen for interictal migraine patients alone ^7^. Schankin *et al*. suggest that visual snow is a unique visual disturbance, distinct from migraine with aura. However, the pathophysiology may be associated with migraine as well ^8^.

## Gastrointestinal disturbance

Gastrointestinal symptoms are common accompanying symptoms of migraine. Nausea occurs in 70–80% ^9, 10^ and vomiting in 30% of migraine patients ^2^. Anorexia is also common in migraine patients. However, because of the different study methods, the frequency of anorexia is variable from less than 20 to more than 82% ^11– 13^. In addition, migraine patients experienced delayed gastric emptying during and outside of a migraine attack ^14, 15^. The head-HUNT study in Norway showed that gastrointestinal complaints increased with increasing headache frequency, including migraine ^16^. In a recent report of the American Migraine Prevalence and Prevention (AMPP) study, persistent frequent headache-related nausea, defined as nausea frequency of more than 50% within 2 years, was common (43.7%) among subjects with episodic migraine. Migraine patients with frequent nausea were twice as likely to progress to chronic migraine (CM) compared to those with no or a low frequency of nausea ^17^. Autonomic nervous system dysfunction is suspected to be related to migraine patients with gastrointestinal symptoms ^18, 19^. In one PET study, activation of the rostral dorsal medulla and periaqueductal gray was noted in patients with nausea as a premonitory symptom ^20^. Anorexia is a major symptom of abdominal migraine, a variant of migraine with recurrent abdominal pain, mainly in children. About 70% of them develop migraine in adults. Abdominal migraine can persist into adulthood in some patients ^21^.

## Allodynia

Allodynia is a painful sensation caused by innocuous stimuli, such as light touch, brushing hair, wearing glasses, and so on. It was reported in 70% of episodic migraine and in 90% of CM patients ^22, 23^ and was associated with anxiety, depression, and disability ^24^. The risk factors of allodynia in migraine patients included obesity, high frequency of headache, and female gender ^25^. Allodynia usually develops within a few hours after migraine attack and is associated with inconsistency of response to triptans from one attack to another within patients ^26^. The AMPP study showed that allodynia was associated with a lower efficacy of acute medications ^27, 28^. ****The poor and inconsistent response to triptans and other abortive medications is explained by the fact that allodynia is the clinical manifestation of central sanitization ^29, 30^. The activation of second-order neurons in the brainstem, particularly the trigeminal nucleus caudalis, is related to cephalic allodynia. If the allodynia is noted in other sites outside of the head, sensitization of the third-order trigeminal neurons in the posterior thalamic nuclei is suspected ^23, 26, 31^.

Two blood oxygen level-dependent (BOLD)-fMRI studies demonstrated abnormal activations in migraine patients with allodynia ^32, 33^. However, the activation sites were not consistent. In one study, the activation of the dorsal pons and contralateral inferior parietal lobule were lower in migraine patients with allodynia ^32^. In the other study, greater activation at the anterior cingulate cortex and middle frontal gyrus was observed in migraine patients with allodynia during moderate-noxious trigeminal heat stimulation (51°C) but lower activation in the secondary somatosensory cortices was seen during high-noxious trigeminal heat stimulation (53°C) ^33^. The authors suspected a dysfunctional analgesic compensatory mechanism and an abnormal internal representation of pain in migraine patients with allodynia ^32, 33^. Very recently, a PET study with [ ^11^C] raclopride found that there is a transient dopamine reduction in the striatum region during migraine attacks and allodynia. However, a relatively sudden increased dopamine release in the insula was noted during allodynia, the late stage of migraine attack. The authors suspected that the reduction and imbalance of dopamine between striatum region and insula account for the pathogenesis of the allodynia ^34^.

Schwedt *et al*. showed that, in addition to allodynia during migraine attack, low heat pain thresholds between migraine attacks were found in migraine patients and the thresholds were positively correlated with the number of hours until the next migraine attack. The authors suspected that the lowered pain threshold was an early sign of a migraine attack ^35^. Similar findings were also found in a small-sized study: the lower pain thresholds were detected during the pre-attack phase of migraine but not during the interictal period ^36^.

## Vestibular symptoms

About 30–50% of migraine patients report vertigo, dizziness, or balance disturbances associated with migraine attack ^37– 39^. The frequency of vestibular symptoms is much higher in patients with migraine than in those with other headache types ^40, 41^.

Several terms have been used to describe vertigo occurring with migraine. The term “vestibular migraine” was co-defined by the International Headache Society and Barany society. The criteria for vestibular migraine are listed in the Appendix of International Classification of Headache Disorders, third beta edition ^42^. Both migraine and vestibular symptoms are very common in the population and therefore co-existence is possible. It is important to note the similarity of migraine with brainstem aura and vestibular migraine. In migraine patients with brainstem aura, vertigo is one of the aura symptoms. Of note, at least two aura symptoms of brainstem origin are necessary to make the diagnosis of migraine with brainstem aura, including dysarthria, vertigo, tinnitus, hypacusis, diplopia, ataxia, and decreased level of consciousness. The auras should be accompanied or followed by the headache within 60 minutes ^42^. The diagnosis of vestibular migraine requires the exclusion of other potential causes of the vestibular symptoms. It usually consists of a detailed history of both headache and vestibular components in addition to potential testing, such as imaging and audiometry. The auditory symptoms, such as hearing loss, tinnitus, and aural pressure/fullness, are common but usually mild in vestibular migraine patients ^43, 44^.

The vertigo symptoms vary in patients with migraine. They can occur spontaneously without any trigger or may be provoked by positional change ^45^. The frequency of spontaneous vertigo seems to be much higher than positional vertigo in patients with vestibular migraine ^45^. In a recent study with a nine-year follow-up, the accompanying vertigo could be persistent during migraine attacks, which caused a severe impact on quality of life. The authors also reported increased interictal ocular motor abnormalities from 16 to 41% at follow-up ^43^.

The pathophysiology of vestibular migraine is still unclear. The activation of vestibular and cranial nociceptive pathways at the same time was suspected to be one possible cause. Recently, several neuroimaging studies support the possibility. One whole-brain BOLD-fMRI study showed abnormal thalamic functional response to vestibular stimulation in patients with vestibular migraine ^46^. In a recent MRI study for structural changes, increased gray matter volume of the left thalamus, left temporal lobe, frontal lobe, and occipital lobes as well as decreased gray matter volume of the left cerebellum were found in patients with vestibular migraine compared to migraine patients without vestibular symptoms ^47^. During the attacks of vestibular migraine, flurodeoxyglucose PET scans demonstrated increased metabolism in the temporo-parieto-insular areas and bilateral thalamic regions. The finding indicates activation of the vestibulo-thalamo-cortical pathway ^48^.

There are only a few studies focusing on the treatment of vestibular migraine ^49^. Patients are often treated with typical migraine prophylaxis. Zolmitriptan was reported to be better than placebo during acute attack of vestibular migraine ^50^. In addition, flunarizine was shown to be an effective preventive agent for vestibular migraine ^51^.

Actually, vertigo is very common in the general population ^52^. The associations between migraine and benign paroxysmal positional vertigo (BPPV) have been reported. One study revealed that migraine was three times more common in patients with BPPV than in the general population ^53^. Another population-based study in Taiwan showed that patients with migraine had a 2.03-fold increased risk of developing BPPV compared with the control group ^54^.

## Restless legs syndrome

The relationship between migraine and sleep has been known for more than a century ^55^. Sleep disturbance is not only a common complaint of migraine patients but also a trigger of migraine attack ^56^. There is accumulating evidence to show that RLS, an important cause of sleep disturbance, is one of the comorbidities of migraine ^57– 64^. Some similarities are noted between migraine and RLS—e.g. female predominant, episodic attacks, and fluctuations in levels of certain biogenic amines during attacks—and are postulated as crucial factors in the pathophysiology ^65– 67^. The frequency of RLS in migraine patients is variable (about fourfold increased odds compared to healthy controls); however, it is lower in Asians but higher in non-Asians. The frequency of RLS in migraine patients did not differ between genders or those with and without aura ^57– 64^.

The pathophysiology of the association is still unknown. However, some clinical findings provide clues. A recent study of a temporal relationship between migraine and RLS attacks showed bidirectional triggering association between migraine and RLS attacks in migraine patients with comorbid RLS ^68^. In addition, migraine patients with RLS were more likely to have photophobia, phonophobia, exacerbation due to physical activities, vertigo, dizziness, tinnitus, and neck pain ^57^. The results suggest that migraine severity is related to RLS frequency and explain the reason why the propensity of RLS is higher in clinic-based studies as compared to community studies ^57– 64^. Valente *et al*. reported a higher familial predisposition to serotoninergic drugs in migraine patients with RLS ^69^. In a genetic study, Fuh *et al*. demonstrated that *MEIS1* variants, a known gene for RLS alone, are also associated with an increased risk of RLS in migraine patients ^70^. All of these reports hint that the balance of dopaminergic and serotoninergic systems could be crucial in the association between migraine and RLS.

## Psychiatric disorders

Migraine is comorbid with many psychiatric disorders ^71^. Of them, depression, bipolar disorder, and anxiety disorder are common and cause disability in affected patients ^72^.

### Depression

Epidemiology studies demonstrate that migraine subjects are at two- to fourfold increased risk of developing lifetime major depression in comparison with subjects without migraine ^73– 75^. The association is stronger in migraine with aura than in migraine without aura ^76, 77^. In addition, CM patients had a higher risk of depression as compared to patients with episodic migraine ^78, 79^.

The pathophysiology of this comorbid association is not known, but genetic factors, serotonergic dysfunction, ovarian hormone influences, and hypothalamic-pituitary adrenal axis dysregulation are suspected to be involved long term ^80^. Lirng *et al*. recently measured brain metabolite concentrations, including myo-inositol (mI) and total creatine (tCr), by MR spectroscopy. They demonstrated that migraine patients with major depression had higher mean mI:tCr ratios in the left and right dorsolateral prefrontal cortex compared to migraine patients without major depression ^81^.

### Bipolar disorder

Migraine patients are reported to have a higher risk of comorbid bipolar disorder ^82– 84^. Bipolar patients are also reported to have a higher frequency of migraine, particularly among bipolar disorder II subjects ^85^. Of note, bipolar disorder relative risk is increased to 3.88 in CM compared to normal controls and 1.81 compared to episodic migraine ^86^. In addition, patients comorbid with migraine and bipolar disorder seem to be more likely to have a rapid cycling illness course ^87, 88^.

### Anxiety

The prevalence of anxiety is about two to five times higher in patients with migraine than in the general population ^89^. Similar to other psychiatric comorbidities, anxiety is much more common in CM patients than in those with episodic migraine ^90^. Alessandra *et al*. recruited 1,261 migraine patients in Brazil and classified them into four groups based on migraine frequency. In comparison with controls, the migraine patients had a higher risk of generalized anxiety disorder and the risk increased with headache frequency. Similar comorbid associations were also noted in panic disorder and obsessive-compulsive disorder ^91^. The risk of panic disorder was also reported to be 3.6 times higher in migraine patients in a meta-analysis from 1990–2012. The presence of panic disorder in migraine patients is associated with more frequent attacks, increased disability, a higher risk for chronification, and medication overuse ^92^. In addition, osmophobia in migraine patients is reported to be associated with significant anxiety symptoms ^93^.

## Conclusions

Migraine is a disabling neurological disorder, not only because of the headaches. Many non-headache symptoms and comorbidities of migraine have a significant negative impact on patients’ quality of life. There are many more studies focusing on this issue. For better quality and strategy of migraine patients’ care, more studies need to be conducted to explicitly investigate these problems from a therapeutic point of view, given the clinical implications.
